# Book Reviews

**Published:** 1809-08

**Authors:** 


### CRITICAL ANALYSIS
### OF THE
### RECENT PUBLICATIONS
### ON THE
### DIFFERENT BRANCHES OF PHYSIC, SURGERY, AND MEDICAL PHILOSOPHY.

---

*Medico-Chirurgical Transactions, published by the Medical and Chirurgical Society of London. Vol. I.*

( Continued from our last, pp. 65—78. )

ARTICLE 8.—*Case of Exposure to the Vapour of burning Charcoal.* By WILLIAM BABBINGTON, M. D. F. R. S. Sen. Physician to Guy's Hospital.

This paper abounds with good observations, many of which are more calculated for the public at large than the medical reader. We therefore trust the whole will be inserted in every popular Journal.

Two of the waiters at a public house in Honey-lane Market, totally unconscious of the danger to which they exposed themselves, went to rest with a chaffing dish of burning charcoal at the foot of their bed. In the morning, not appearing at the usual hour, they were called by a person who found one of them lifeless on the floor. The other was insensible, and apparently at the point of death, but with perceptible breathing, heat, and pulsation at the arteries. The situation of the first being the most urgent, Dr. Babbington tried the effect of artificial respiration, by instructing instructing Mr. Hingeston to inflate and empty the lungs; Mr. H. blowing through a catheter, passed by the mouth into the trachæa, and his assistant alternately pressing out the air.

As all this proved ineffectual, the Galvanic apparatus was introduced; and the muscles showing no irritability to this powerful stimulus, it was reasonably supposed that all further attempts would prove ineffectual.

In the elder waiter there was stertorous breathing, countenance somewhat flushed, pulse full and strong; it was therefore thought adviseable to bleed him. But upon returning to him, it was found that the vital powers were much more enfeebled than before; the pulse was weaker, and the respiration more imperfect; the heat confined to the upper part of the body; the eye insensible to common impressions; and a slight convulsive motion, which some of the muscles had before exhibited, had ceased; the tongue swoln and projected from the mouth, which was locked by a spasmodic action of the muscles of the lower jaw; there was a copious discharge of frothy saliva from the angles. Under these circumstances the galvanic shock produced instantly a good effect, affecting not only all the muscles through which it was immediately passed, but most others subservient to the functions of life as well as to the organs of sense.

About two hours after the event was first discovered, a bladder of oxygen gas was procured, the inspiration of which seemed to be followed by an increased activity in respiration and arterial action. As the heat of the body was not deficient, the face and chest were sprinkled with cold water, which, adds the author, "had also the effect of rousing the dormant powers of sensation, as the respiratory muscles were uniformly thrown into action, though in a more feeble and interrupted manner than when we employed the galvanic influence." These processes of galvanism and oxygenation were alternately employed every half hour for two hours and a half more, when the galvanic application was discontinued, as the heart, though always excited by it, seemed in the intervals to act more feebly, and to endanger a destruction of that equilibrium of forces which is necessary to the maintainence of life. Hartshorn was rubbed on the temples, and the lower extremities being cold, bottles filled with warm water were applied to the feet, whilst the trunk of the body was covered with bed clothes. In an hour after, a gentle perspiration was perceived over the whole body; the pulse fuller; the respiration easier. The inhalation of gas was continued however at longer periods for three hours more. The spasm on the jaw having subsided, the patient swallowed liquids in small quantities without apparent difficulty. The pulse now rose to 120 with some strength. About this time the ligature slipped from the arm; a pound of blood escaped, which produced at first alarming symptoms of debility. During the night however the strength recovered. On the following day symptoms of paralysis appeared, but gradually passed off.

Some very ingenious reflections follow by Dr. Babbington on the cause of the deliterious property of carbonic acid gas. Rozier and Davy conclude from their experiments, that in its undiluted state it is wholly irrespirable, and produces a similar spasm on the epiglottis as if the animal were immersed in water. If this be the case, says Dr. Babbington, how shall we explain the loss of muscular irritability in animals destroyed by this gas? This is a very just inquiry; but we submit it to that ingenious physician, whether a sufficient number of experiments have been made in destroying animals by gases, to determine the uniformity of such consequences as he describes. Under some circumstances of drowning, it was observed by Mr. Hunter, that what he calls absolute universal death followed. In these cases, of course, there could be no more irritability in the muscles than in the unhappy subject, who could not be roused from the situation in which he was found, and whose severer fate is very rationally imputed to his having fallen on the floor, by which he was still more exposed to the effects of the carbonic acid gas, whose specific weight is known to be greater than that of common air.

Respecting the mode of treating such cases, we cannot but agree with Dr. Babbington, that where respiration is suspended, or even impeded, our first object must be to restore it artificially; and if the appearances he describes have uniformly shown themselves in dissecting animals with a double heart, which have died from this cause, there can hardly be a doubt that death is occasioned by a spasm on the epiglottis. For in common cases of sudden death, where all the parts have immediately ceased to act, the left side of the heart, and the arteries, are found filled with blood; whereas where death has gradually followed the loss of that series of actions by which life is supported, the last act has usually been the muscular contraction of the arterial system by which the blood has been forced into the veins. Unfortunately, the friends of the boy could not be prevailed upon to suffer the body to be opened. It might have afforded some information had it been recorded, whether stiffening came on after death; whether blood could be drawn from a vein; and if drawn from any part, whether it had the power of coagulation.

That the boy's case was hopeless, however, appears not only from the event, but from the inefficacy of every attempt made towards resuscitation. We shall therefore offer only a single hint on the situation of such as may be found in the act of breathing, however laboriously or imperfectly. And first, after a removal to a purer air, we cannot help suggesting the importance of sprinkling with cold water till such other means are procured, as in this case were found useful. We should also advise the utmost caution in suffering the body, particularly the lower extremities, to lose the heat it has retained. Sprinkling with cold water has been found sufficient of itself in some very urgent cases, which, as far as our memory serves, were not less serious than that of the elder waiter.

We

We refer to one mentioned in a volume of Dodsley's Annual Register, which may be easily referred to, as it was recorded before the Index was published to that valuable work. Most probably the Gentleman's Magazine would furnish the same.

The other means suggested by our author, are such as we cannot but approve. But we must offer a hint, which we have found almost innumerable occasions to enforce in sick chambers. When Dr. Babbington's patient recovered his reason, the first thing that distressed him was to find himself in a strange place. From the period that he fell asleep, to the time we are now describing, he had been perfectly unconscious of his own existence. Had he awoke to the scene he had left, how much distress might he have escaped, which, though of short duration, was at a most interesting period. In many cases of fever, the moving a patient's bed, whilst the actions of the brain have been vacillating, has been followed by terrors, which the state of his mind rendered it impossible to relieve.

ARTICLE 9.—*Case of Lithotomy, with Remarks.* By THOMPSON FOSTER, Esq. Surgeon on the Staff of the Army, and Sen. Surgeon to Guy's Hospital.

This paper contains some judicious cautions on selecting the proper state of a patient for performing this important operation, and particularly of admitting no delay whenever such a state occurs.

ARTICLE 10. — *On Gouty Concretions, or Chalk Stones.* By JAMES MOORE, Esq. Surgeon to the Second Regiment of Life Guards.

A good sytematic description is here given of the process of Nature, in the deposition of gouty calculous matter, and also the means by which she disengages the parts from it. Some useful practical remarks follow, on the mode of treatment under cases of particular urgency.

ARTICLE 11.—*Case of Artificial Dilatation of the Female Urethra.* By H. L. THOMAS, Esq. F. R. S.

This paper contains two very interesting cases. In the first, as mentioned above, the urethra was in a very short time so dilated, that the operator introduced with ease his finger and thumb, and relieved his patient of an ivory ear picker, which, whilst it was used by her husband to remove a slight difficulty in making water, had been suddenly retracted into the bladder. No incontinence of urine followed more than six hours afterwards. Mr. Thomas's success induces him to propose, that in all cases of stone in the female bladder, recourse should be had to dilatation, rather than a section, which as frequently ends in an incontinence of urine as the greatest and most continued dilatation. The other case, tho' it could not be more successful, is perhaps more creditable to the chirurgical dexterity of the author, from the greater difficulty and the novelty of the attempt. We cannot but transcribe it in his own words.

 "A gen-

"A gentleman of an inactive and sedentary disposition had for many years suffered from contispated bowels, which increased to that degree that the most active cathartics failed in producing the desired effect. By the advice of a practitioner, whom he consulted in Paris, he daily introduced into the rectum a piece of flexible cane (about a finger's thickness), where it was allowed to remain until the desire for evacuating the fæces came on. This plan succeeded so well that for more than a twelvemonth he never had occasion to resort to any other means. One morning, being anxious to fulfil a particular engagement in good time, in his hurry he passed the stick farther up, and with less caution than usual, when it was suddenly sucked up into the body, beyond the reach of his fingers. This accident, however, did not interrupt the free discharge of the fæces, and the same evacuation regularly took place every day, whilst the stick remained in the gut. It was seven days afterwards when I first saw him; he was in a very distressed state, with every symptom of fever, tension of the abdomen, and a countenance expressive of the greatest anxiety. His relatives and friends were totally ignorant of the real nature of his case; and nothing less than the urgency of his sufferings, could ever have prevailed upon him to disclose it to me. Such were his feelings on the occasion, that a violent hysteric fit was brought on by the mere recital of what he termed his folly.

"Upon examination with my finger, per anum, no part of the cane could be discovered; but one end of it was readily felt projecting (as it were) through the parietes of the abdomen, midway between the ilium and the umbilicus on the right side. The slightest pressure upon this part gave him exquisite pain.

"After repeated trials I was at length enabled, with a bougie, to feel one extremity of the stick lodged high up in the rectum; but without being able to lay hold of it with the stone forceps. To allay the irritation for the present, an emollient clyster, with tinct. opii. ʒij. was given, which passed without the least impediment, and did not return. On the next examination, two hours after, I found the sphincter ani considerably dilated, and by a continued perseverance to increase it, the relaxation became so complete, that in about twenty minutes I was enabled to introduce one finger after the other, until the whole hand was engaged in the rectum.

"I found the end of the stick jammed in the hollow of the sacrum, but by bending the body forward it was readily disengaged, and extracted. Its length was nine inches and a half, with one extremity very ragged and uneven.

"For several days after, the situation of the patient was highly critical; the local injury, joined to the perturbation of his mind, brought on symptoms truly alarming. At length I had the satisfaction to witness his complete recovery; and he has ever since (more than two years ago) enjoyed good health, and the regular action of the bowels, without the assistance of medicines, or any other aid."

ARTICLE

ARTICLE 12.—*Case of Hydrophobia, with an Account of the Appearances after Death. Communicated by* ALEXANDER MARCET, M. D. F. R. S. one of the Physicians to Guy's Hospital.

This account cannot be expected to furnish much novelty in this melancholy disease; it is however very well worth being recorded, for no facts can be unimportant in a malady that baffles not only all our remedies but all our researches.

There are two or three particulars worthy of notice. The first is, that the dog appears to have been diseased, refusing his food, and barking less distinctly than usual. He left his home, as has always been remarked, and as far as can be judged, died without any appearance of violence. He bit his master in two places, who without using any precautions, remained well.

The patient lived to the sixth day after the first appearance of the symptoms. The pharynx was found inflamed, and inflamed portions of the œsophagus appeared also. The vessels on the surface of the brain were turgid; some of the vessels of the pia mater contained small bubbles of air.

The remedies used were opium to a considerable quantity, though, as the writer expresses himself, without much hope of success. Iron and arsenic had also a full trial. Hyocyamus seemed to exasperate the symptoms, but this inference must be uncertain.

" I would also (says our author) beg leave to point out a circumstance which does not seem to have been sufficiently remarked; I mean the pain which is felt in the parts contiguous to the bitten spot, at the moment the symptoms of hydrophobia are developed. This pain, it would appear, is apt to follow the course of the nerves rather than that of the absorbents. In the present case,* as well as in one of the cases detailed by Dr. Babbington, there was pain in the arm and shoulder, but without any affection of the axillary glands; and in another case, published in the second volume of the *Medical Communications*, the pain occasioned by a bite in the leg was referred to the hip and loins, without any affection of the inguinal absorbents. Should this remark appear to have any weight, the consequence which would naturally be drawn from it would be, that when the precaution of removing or cauterizing the part has been neglected in the first instance, it may still be adviseable to have recourse to it at any subsequent period before the developement of the disease."

We believe the late Mr. Hunter once entertained this expectation of excision at any period before the symptoms appeared, but in one attempt found himself foiled. We shall be glad if those who have had larger opportunities would correct us if we are mistaken, or add to our information if right.

* " We dissected and examined the radial nerve; but, as we fully expected, not the least diseased appearance could be discovered."

ARTICLE 13.—*Account of Three Cases of sudden Death, with the Appearances on Dissection, and some additional Observations, by* THOMAS CHEVALIER, Esq. F. R. S. Surgeon to the General Westminster Dispensary, and Surgeon Extraordinary to the Prince of Wales.

In these three cases no appearances of disease could be discovered; but the heart was in all found destitute of blood, yet not contracted. A similar case is extracted by our author from Bonetus, and another from Morgagni. Some judicious observations follow of Mr. Chevalier's; and two cases are related by Mr. Wood, in which the previous symptoms were somewhat analogous, but relieved by vol. alkali and other stimulants.

ARTICLE 14.—*Case of Intus-susceptio, with Remarks.* By THOMAS BLIZARD, Esq. F. R. S. Surgeon to the London Hospital.

Our readers will recollect, or may turn to a paper in our Journal, No. 35, for January 1802, by Dr. Hull, of Manchester. In this the ingenious author (page 32), refers to a paper in the second volume of Transactions of a Society for Medical and Chirurgical Improvements, where a substance is described resembling gut, about a yard long, discharged by a patient under symptoms of colic and dysentery. This substance being accurately examined by Dr. Baillie, was admitted to be a portion of colon. In this Dr. Hull is ready to agree; but these two physicians differ in the probable manner in which the continuity of the intestinal canal was preserved after so large a defalcation. Dr. Baillie conceived that under high inflammation, ending in mortification, coagulated lymph was thrown out, surrounding the inflamed part, and when the mortified part was separated, that the cavity was preserved by this coagulated lymph, which retained the tubular form of the intestine it had before surrounded. Dr. Hull, whilst he admits the advantages Dr. Baillie has over him, from his extensive opportunities of seeing diseased dissections, expresses his dissent with much modesty; and from analogous cases which were examined after death, and are recorded by different writers, does not scruple to give it as his opinion that the disease was *Intus-susceptio*; that the incarcerated part mortified at its extremities was cast off, and that the continuity of the canal was preserved by an adhesion at the divided extremities of the containing gut, with the parts immediately contiguous. In the succeeding Number of our Journal, is contained a most valuable, though short paper, from Dr. Baillie, in which that gentleman, with a candour which distinguishes his every transaction, makes no scruple to admit the greater probability of Dr. Hull's opinion, particularly as it is supported by cases strongly analogous, and in which the parts were examined after death.

The case contained in Sir W. Blizard's paper, now in order before us, is of a child, who after the usual symptoms of constipated bowels, died. On dissection, six inches of the intestinum ileum, the cæcum cæcum with its appendix, and ascending colon with its transverse flexure, were found compressed within the sigmoid flexure of the colon extending to the rectum. The rest of the intestines were little altered, but the intus-suscepted parts were in a complete state of strangulation, and black. "Had the child's constitution," continues Sir William, "been able to sustain the separation, inflammation might no doubt have produced an union of the ileum with the lower part of the colon; the continuity of the canal would thus have been preserved, the separated part might have passed with the fæces, and the child have recovered."

Sir William then refers to Dr. Baillie's paper, and makes the same apology for differing from him, that we have seen made by Dr. Hull. In a note it is added, that he, (Sir W.) was not aware, when he wrote his paper, that Dr. Hull had in the Medical and Physical Journal, "adopted a similar view of the point in question."

On this occasion we cannot help remarking, that for some years past, it has been too much the custom of metropolitan practitioners to undervalue *medical reading*. Perhaps Mr. Hunter may be chargeable with first introducing, by countenancing this fault. It is well known that gentlemen *read* but little; and if any other physiologist should *do* as much as he has done, perhaps we may make the same excuses for him. However, justice to that celebrated character, obliges us to remark, that whenever he wrote, he has shown himself careful to collect the opinions of his predecessors. It must be admitted, that for men in full practice, this would be a difficult task, and perhaps, if it were always required of them, we might lose many valuable facts and observations which we now possess in a crude state. Nor can we, on this occasion, fail to mention the advantage which medicine has derived from our hazardous, but as it has proved, fortunate undertaking. By means of our Journal, the most important facts have been not only circulated several years sooner than they could otherwise have been generally known, but any inaccurate conclusions have been noticed; and as we see in the remarks of Drs. Baillie and Hull, corrected with equal celerity, and perhaps with greater precision than any other means could have accomplished.

Article 15.—*Description of Two Muscles surrounding the membranous Part of the Urethra.* By James Wilson, Esq. F. R. S. &c. and Vice President of this Society.

"In my lectures on the organs of generation, I have, for these last ten years, demonstrated to the students attending in Great Windmill Street, two very distinct fleshy bellies belonging to muscles of a triangular shape, united below by one common tendon, but each having a separate tendinous attachment to the inside of the symphysis of the pubes, and which are so placed as to surround the membranous part of the urethra. The tendon belonging exclusively to each muscle, is at first of a round shape, but soon becomes comes flattened as it descends; it is affixed to the back part of the symphysis of the pubes; in the adult, about one-eighth of an inch above the lower edge of the cartilaginous arch of the pubes, and nearly at the same distance below the attachment of the tendon of the bladder; to which, and to the tendon of the corresponding muscle, it is connected by very loose cellular membrane. The tendon descends at first in contact with, and parallel to its fellow; it soon becomes broader, and then sends off fleshy fibres, which also increase in breadth, and when near to the upper surface of the membranous part of the urethra, separate from those of the opposite side, spread themselves on the side of the membranous part of the urethra through its whole extent, then fold themselves under it, and meet in a middle tendinous line, with similar fibres of the opposite side. One extremity of this common tendon is connected with the posterior part of the tendon joining the acceleratores urinæ muscles, and which, in the perinenm, joins also with some of the fibres of the sphincter ani and transversales perinei muscles.

"The line of tendon connecting the two bellies of these muscles, is in general very distinctly seen running from the apex of the prostate gland, along the under surface of the membranous portion of the urethra, until it enters the corpus spongiosum penis. Sometimes, however, it is more faintly marked, and the flehy fibres then appear to be continued into each other."

One effect of the attachment and course above described, the author very reasonably remarks, must be to draw the membranous part of the urethra upwards, so as to compress it against the inside of the cartilaginous arch of the pubes, another to diminish or even close the urethra at that part.

"It is well known that the part where the membranous portion of the urethra joins the penis, is naturally the narrowest part of the canal; it is at this part that the chief impediment is felt, in irritable urethræ, to the introduction of any instrument; and here strictures generally begin to be formed. The contraction of these muscular fibres must occasionally increase the difficulty, and sometimes of itself produce it. When bougies have been introduced into very irritable urethræ, and have been permitted to remain in them a few minutes, I have often observed, on their being withdrawn, that they were much flattened at that part which lay in the membranous portion of the urethra. This could only be occasioned by the muscles, which in the perineum are connected with the middle tendon of the muscles now described, contracting at the same time, rendering the perineum a fixed point, and thereby obliging the fibres of the muscles surrounding the urethra, to form in their contraction a straighter line, and thus to compress the sides of the urethra more than the under part, and by this means to change the urethra from a circular to an elliptical form.

"Knowing," concludes our author, "that such muscles exist, we shall not hastily infer, that a permanent stricture of the urethra is formed, because we cannot immediately introduce a bougie or catheter.

catheter. We shall therefore avoid using those remedies, which, though adapted to the cure of a stricture attended with a morbid alteration of the urinary passage, would, in a case arising from contraction only of these muscles, prove injurious to our patient. In cases of retention of urine, where no instrument will enter the bladder, we shall be induced to persevere in the means best adapted to overcome a temporary but forcible contraction of muscles, which is constant, but which are seldom thrown into such strong action. When this has been done, our second attempt to draw off the urine will probably succeed. It is my wish here, however, to point out an arrangement of muscular fibres till now overlooked, not to reason on such arrangement."

This truly valuable paper is accompanied with an engraving to illustrate it. We shall only remark, that the sphincter vesicæ, and the muscular fibres of the urethra, are more easily talked of than demonstrated. The muscles above described cannot hereafter escape the notice of any diligent anatomist, and every practical surgeon will keep them in view, whenever he meets with difficulties in passing an instrument of any description, especially in that part of the urethra.

Article 16.—*Case of Tumour in the Brain, with Remarks on the Propagation of Nervous Influence.* By John Yellowly, M. D. Physician to the London Hospital.

This case furnishes the basis of a very long dissertation, we shall give it therefore at full length, as the text of the paper.

" David Thomas, a man of a fair complexion, and of about thirty-six years of age, became my patient in the General Dispensary in December 1806, on account of a slight paralysis of the right side, and a distortion of the left eye. He had been subject, for twelve months before, to occasional severe attacks of pain of the head, shooting from behind forwards; and about six weeks previous to my seeing him, he was surprised, on awaking in the morning, to find his left eye drawn inwards, and his vision double. In two or three days more, his right hand became weak; and this was gradually followed by weakness, and afterwards by numbness of the corresponding leg and side; and by a slight stammering, and a small degree of distortion of the mouth.

" These symptoms continued when I first saw him, with some degree of head-ache, and his pulse about sixty-eight, and rather weaker in the affected than the sound arm. In other respects he was in his accustomed state of health. The left eye was drawn towards the nose, but the pupil was in its usual state of sensibility to light. The double vision continued. All voluntary power over the abductor muscle was lost; nor did the affected eye, as in common cases of strabismus, recover its usual position on shutting the sound one. He had been purged and blistered by a gentleman well versed in the treatment of complaints of the eye, when the distortion tortion first came on; but he ceased to be under his care on the paralysis supervening.

"In little more than a week from the time of my first seeing him, he became at first slightly, and then considerably affected with convulsive motions of the whole body. These recurred at more and more frequent intervals, he became gradually less and less sensible, and died in about twenty-four hours from their commencement. I saw him a few hours previous to his death. He was then in a state of insensibility, with his eyes suffused, his pulse weak, frequent, and fluttering, and his respiration laborious. The distorted eye had recovered its usual position a few hours before, and the pupils were insensible to the action of light.

"On dissection, the brain was found to be of an unusually firm texture, with about half an ounce of water in the ventricles. There was no diseased appearance in the right side of the head, but in the left, a tumour was discovered on the tuberculum annulare, which my friend and colleague Mr. Thomas Blizard, surgeon to the London Hospital, did me the favour to examine with me. 'It was about the size of a hazel nut, and was lying on, and sunk into the tuberculum, at its posterior part, on the left side. It extended to the corpus pyramidale of the same side, pressing upon, and entirely obscuring the left abductor nerve. The tumour was closely connected with the basilar artery, half an inch from the union of the vertebrals to form it; and the coats of this artery had become so tender, that they readily gave way from the application of a probe, which passed through the tumour. The tumour was in a state of imperfect suppuration, and a small coagulum was formed on the diseased part of the artery, similar to what is found in ancurismal arteries.'

"The tumour now described, agreed very much in nature with those, which various authors on the subject of morbid anatomy have mentioned, as being occasionally found in the brain. It seemed to be of a scrophulous nature, and its appearance and imperfect suppuration were analogous to those of scrophulous tumours, formed on the surface, or in any of the cavities of the body. The pressure which it made on the tuberculum annulare and medulla oblongata, there appears to be no doubt, gave rise to the pain of the head, the strabismus, the gradual production of paralysis, and the convulsions which occurred in the latter period of the patient's life. The distortion of the left eye towards the nose, was the necessary consequence of the nerve being affected, which gave energy to the abductor muscle of the eye; and as it arose from the preponderating influence of its antagonist, the adductor, it went off at that period, near the close of life, when the whole nervous system became in a great degree inert.

"The pressure which was made on the basilar artery, had produced a considerable thinness and tenderness in its coats, so as to make them readily give way, on the application of a probe. Had this gone on a little further, the patient must have lost his life by the the vessel giving way, from its inability to resist the pressure of the column of blood which passed through it."

The combined circumstance of the paralytic symptoms on the *right* side of the body, and in a muscle of the *left* eye, apparently arising from a compression on the *left* side of the brain, have led the author into an enquiry concerning the propagation of nervous influence from the brain and spinal marrow, to the muscles of different sides. The structure of the spinal marrow and the effects of injuries it sustains, are first considered according to the opinion of different anatomists.

Haller, it is observed, in one part of his writings, speaks of the same side of the body on which the spinal marrow has suffered an injury as becoming paralytic; in another place it is shown, that he describes the opposite side from that in which the spinal marrow has been affected as paralyzed. From hence our author concludes, that Haller's remarks are not founded on his own observations.

Soemmering observes with the former writer, a decussation of medullary fibres in the medulla oblongata, immediately below the ninth or lingual nerves. He speaks of injuries to the spine as affecting the side of the body, in which the injury has happened; yet, in another passage, he conceives it highly probable that the fibrils of the spinal nerves, every where belong to the side of the body, opposite to that in which they are dispersed.

Sabatier merely describes the two columns of which the spine is composed.

Vicq d'Azyr's remarks go principally to show a communication between the two columns into which the medulla spinalis is divided.

Having shown the difficulty of learning any thing from the physiological speculations of former writers, Dr. Yellowly proceeds to inquire how far experiments, observations made in disease, or the structure of analogous parts in other animals may direct us.

Galen, it is observed, gives what appears the result of experiments, by which it was found that if the spinal marrow was divided longitudinally, none of the nerves on either side were paralyzed. If transversely, that then the nerves only directly below the division were paralyzed. On this the ingenious author of the paper very justly remarks, that if there really exists a decussation of nervous filaments from one side to the other, a wound either longitudinal or transverse, however partial, must wound either the origin or ramifications of the nerves on both sides, and consequently should produce paralytic symptoms on both sides.

Some instances are cited of natural structure in some animals, and peculiarities in individual human spines, in which water has been found in a species of canal in the middle of the spinal marrow. Under such circumstances it is remarked, that if there really existed a propagation of nervous influence by decussation of nervous fibres, it must have been interrupted. In insects it is found that the two sides of the medulla oblongata are separate throughout their their whole extent. All these facts and analogies seem to contradict any decussation of nervous fibres from one side to the other.

It is next remarked, that some instances have been known of persons wounded in the spinal marrow, who have lived for a considerable time without any paralytic affection. In such cases, it is added, that probably the medulla spinalis was divided longitudinally and not transversely. Before we offer our opinion on this question we must be satisfied of the fact, for we are quite of opinion with our author, that "it is difficult [not merely] on the principle of decussation, [but from every fact we have witnessed] to conceive a wound in the spinal marrow in any direction, which would not have the effect of dividing a part of the medium of nervous communication."

A case is mentioned, witnessed by several medical gentlemen, in which a tumour, found after death, pressing on the *left* side of the dorsal vertebræ, produced several anomalous symptoms and severe pains on the *left* side of the abdomen, and, a short time before death, paralysis greater on that side than on the right.

To ascertain how far Galen's account might be depended on, Mr. Astley Cooper was prevailed upon to divide one half of the spinal marrow of a dog transversely. The division was made on the right side. The animal at first appeared dead and rigid. In the course of a few minutes he recovered so far as to breathe, but the elevation and depression of the ribs seemed confined to the *left* side. When laid on his back, it was found that the extremities on the *left* side were stiffer than those on the right, and that on bending them they immediately recovered their position. The extremities on the right side were more flaccid, and remained in the posture in which they were placed.

When the animal turned himself, it was entirely by the exertion of his *left* side. When he attempted to stretch himself, he succeeded on the *left* side: he barked slightly. The muscles of his head and the organs of sense seemed to retain their power; he appeared to swallow. The flaccidity of the side on which the operation was performed seemed to increase, but he recovered the power of moving his tail. On the following morning he had convulsions in both, but principally in the wounded side. On examination after death, slight marks of inflammation appeared, and the operation was proved to have been accurately performed. This experiment confirms one part of Galen's assertions, viz. that wounds in the one side of the spinal marrow affect the same side of the body or extremities.

The similarity of structure, observes Dr. Yellowly, in the medulla spinalis and oblongata, renders it probable that the same laws obtain in both. But some experiments of M. Lorry seem to prove, that a wound in one side of the medulla oblongata produces paralysis in the other. Objections are, however, made to M. Lorry's experiments, both on account of the natural structure of the animals (pigeons) on which they were made, and also from the difficulty of conducting such experiments with sufficient accuracy. As it is found that the tongue, the velum pendulum, and occasionally occasionally the muscles of deglutition, are affected in paralytic cases on the same side as the other voluntary muscles, it is conceived a fair pathological inference, that the medulla oblongata and spinalis follow the same law; that is, the parts are affected on the same, not on the side different from that on which the injury is received.

The effects of pressure on either hemisphere of the brain, has been since the days of Hippocrates, for the most part, admitted to produce paralysis on the opposite side. Whether the same takes place in the cerebellum is not so easily ascertained, as that part is rarely injured without so much mischief induced in many parts of the brain, as to preclude any certain conclusion.

Having made these general remarks, our author is lead again into the inquiry concerning decussating fibres, and the result is about as uncertain as before. Much information, it is remarked, might be gained by a minute attention to the effect which pressure in particular parts of the brain might produce on particular nerves. We heartily agree with this proposition, and sincerely wish that this paper was enriched with more experiments than we find it. By these only can we arrive at any explanation of "an unaccountable circumstance" remarked by Dr. Y. that the senses are in general but little affected in this complaint. Even those nerves which are concerned in mere motion, as the 3d, 4th, and 6th pairs, which go to the muscles of the eyes, have their power but little affected." These and many other apparent paradoxes remarked in this paper, can only be solved by repeated and very accurate experiments.

"The brain," continues Dr. Y. "has a remarkable power in accommodating itself to gradual derangement; and in cases where deposition has been made, or growth taken place by slow advances, the act of absorption on the substance of the brain itself, seems to keep pace with the alteration of circumstances, and thus to prevent any undue or irregular pressure."

Yet it must be remembered, that the case related in the beginning of the paper, and which has given rise to those inquiries, seems to have been a gradual formation, and to have proved fatal before any considerable enlargement occurred.

"It seems to be a salutary provision of Nature, that while those parts of the body which are concerned in voluntary motion, are affected in paralysis, through the medium principally of the spinal nerves, those which supply organs immediately concerned in the preservation of life, are connected as well with the brain as with both sides of the spinal marrow. The great sympathetic nerve, by this connection, and by the number of ganglions which occur in the course of it, (which seem to afford an additional source of nervous influence) is well adapted for preserving, in the vital organs, a species of security against that partial interruption to the propagation of nervous influence, which takes place in paralysis."

Yet we find in Mr. Cooper's experiment on the dog, the muscles of respiration, a function most important to life, were very materially terially affected by the operation. At the conclusion of the paper, notice is taken of Drs. Gall and Spuzheim's memoir, of which we gave an account in one of our former Numbers, and the passage quoted in which those gentlemen remark the communication by means of nervous filaments between the two cords into which the medulla oblongata is separated. In answer to this, it is very justly remarked, that this proof of decussating fibres will not materially affect the reasoning.

"If we find (says our author) that pressure on the opposite of the brain will affect such nerves as are sent out from the medulla oblongata, above the place where this appearance of decussation is seen, it seems to be evident, that the decussation occurring at this place, and confined to a very small portion of the thickness of the whole column, is not the means by which the phenomenon is produced.

"Should the portio dura of the auditory nerve be affected in paralysis of the face, additional force is given to this argument; since it has been proved, both by the authors of the memoir, and by the committee to whom we are indebted for the report, that this nerve arises from the medulla oblongata, near its union with the pons Varolii, and not from the crus of the cerebellum."

We have endeavoured to do justice to this paper, on account of the evident pains which it must have cost the author, as well as on account of the obscurity of the subject. But, we cannot help again recommending *agere plus quam scribere.* By experiments well conducted we may determine something; by reasoning without them, nothing; and though quotations from Aretæus and Galen may show the learning and industry of a writer, they at the same time show the slow progress of the art, if we are to depend on writers of that date for facts which we can ascertain ourselves.

ARTICLE 18.—*Case of a Fœtus found in the Abdomen of a Boy.* By WILLIAM GEORGE YOUNG, Esq.

Of this very curious case, we shall only remark, that the subject containing the fœtus was born apparently in good health; that he lived about nine months; during which time every circumstance proves that the fœtus lived and grew. The latter was imperfect in so many respects that it could not have existed an hour, removed from its nidus. In other words, it had no means of supporting its own existence, which was only preserved by communicating vessels with its brother.

A fact so well ascertained, may induce us to believe a story long current in France, and within these few years repeated in one of their Journals, of a youth who lived to the age of puberty with a brother gradually increasing in his abdomen. These cases are very curious; but as they never can furnish any practical information, 'tis enough that they are recorded.

( To be concluded in our next. )

A Treatise

*A Treatise on local Inflammation, more particularly applied to Diseases of the Eye, wherein an Improvement in the Treatment of those Diseases is recommended.* By J. B. SERNY, M. D. Oculist. 8vo. pp. 123. London, 1809.

THE practice of separating the several branches of medicine and surgery appears to be gaining ground in the more civilized parts of the world; though, doubtless, there will always be found some practitioners in all countries who will not scruple to profess every branch. Such a separation of the science is well calculated to promote the improvement of it, and this has actually been found by experience to be the case. The public at large, as well as the profession itself, always give a marked preference to those who confine themselves to particular lines of practice. We remark this more especially in diseases of the Eyes and Teeth; and in those cases where great dexterity of operation is required. Hence arises the rational preference given to hospital surgeons in the performance of the great operations of surgery.

As the organs of vision are the most wonderful parts of our frame, and the instruments of our most important sense, so the diseases of them have always formed a very important branch of medical practice. Our author appears to have devoted much attention to the subject which he has chosen for the exclusive line of his practice, but we think he has been unnecessarily anxious to establish his theoretical opinions. Medical theories are not now the order of the day. The systems of Galen, Stahl, Hoffman, Cullen, Brown, or Darwin, are little regarded by the generality of practitioners, and Dr. S. can scarcely expect better success than these popular theorists.

He commences his Treatise with the theory of local inflammation; and in order to simplify the subject as much as possible, he assumes the inflammation consequent upon a slight bruise, and examines what takes place in and near the part injured. In order to develope the proximate causes of this affection, (for he rejects the common notion of one proximate cause, as being insufficient to explain appearances) he enumerates the following symptoms or events, as taking place in a simple contusion, viz. redness, throbbing, heat, swelling, pain, and various kinds or successions of discolouration. His manner of explaining or accounting for these events, as well as for those which take place in the progress of the cure, constitutes his theory, and is the most original part of his work. As it appears to him to be of great practical importance to elucidate his ideas on this subject fully, he has devoted no less than forty-four pages to it; and after a particular explanation of each part, he recapitulates the substance of his theory of inflammation, by whatever cause produced, to the following import. "A local inflammatory affection occasioned by cold, checked perspiration, violent exercise, &c. is at first produced by an increased impetus impetus of blood to the part, which is become vicarious, for the time, of the whole system, (in this respect differing from those inflammations occasioned by external applications or violence) and this inflamed part, if restored to health by resolution or absorption, will run the following course, in about fourteen stages or different actions. 1st. An increased impetus of blood to the part to be inflamed. 2d. Congestion there in consequence. 3d. Laceration of minute vessels, which is the origin of the pus. 4th. Effusion and swelling in numerous incarcerated cavities. 5th and 6th. Intercepted circulation, with an increase of heat. 7th. The vicarious actions of enlarged and elongated vessels, with redness round the part. 8th. The contraction and healing of the lacerated vessels. 9th. The absorption of the serum of the blood effused. 10th. The death and decomposition of the effused and incarcerated red globules. 11th. The absorption of the same when pus is not formed. 12th. The gradual contraction and obliteration of the vicarious vessels near the part affected, and the disappearance of the redness. 13th. The reproduction of entirely new vessels; and, finally, the complete restoration of the circulation, and the perfect cure of the inflammation.

"The intercepted circulation (5) and increased heat (6) are multiplied or augmented in the very same parts, by two causes. The first, by the vessels becoming impervious through a loss of continuity or laceration of their extremities, producing an effusion and intercepted circulation: and, secondly, this effusion occasioning an additional compression on other minute arteries circumscribing it, and further interrupting the course of the arterial blood in this part.

"The 7th, 8th, 9th, 10th, and 11th stages of local inflammation, from whatever cause, may take on the absorptive or suppurative action; although they are both nearly the same internally, yet forming some external distinguishing characteristic appearances. I mentioned above the absorptive, but in the full suppurative, instead of the lacerated vessels continuing to form many small, detached, incarcerated cavities, containing their respective quantity of effusive fluid, they soon communicate with one another, and forming at last one common, large, swelled cavity. Thereby, during that action, increasing the effusion, and laceration, with increased pain: shewing all the symptoms of a circumscribed inflammatory tumour. The solids of the internal surface of which tumour, undergo several actions or changes, and so also do its contents (5, 6, 7, 8, 9, 10); and upon which internal surface, new vessels spring up called granulations, in proportion as the vicarious vessels are obliterated. So that these new granulations, by becoming very numerous and thick, force the contained fluid in the cavity, called an abscess, to break through the external integuments which have lost their texture. And these granulations (13) continue, and must continue, till the complete obliteration of the vicarious vessels (12) has been perfected; and thereby the the circulation through the injured part restored (14). The circulating equilibrium will then be re-established, and the cure completed, as far as the nature of the injury committed will permit."

Here the author ends his abridged Sketch of the 'proximate and accessory' causes and stages of inflammation; and he contends, that in the cure of such affections, it is of material importance to keep these several actions or events in view. This he exemplifies in the treatment of the inflammations of the eye in the practical part of his work, which immediately follows.

The diseases which he has selected are,—1. *Ophthalmia*; in the treatment of which, besides the usual antiphlogistic plan, scarifications, &c. he recommends two very different external applications during the night and the day. The nightly cataplasms are only to be continued till the tension, pain, and throbbing are relieved, which he observes they do by their anodyne and emolient powers. The other external applications are, mild tonic lotions, employed frequently, and cold; the light being partially excluded by a shade during the day.

"Let this remark," he says, "be particularly remembered, that whoever has his eye or eyes covered up day and night, even for a short time only, may have the transparency of the *cornea* destroyed, by the matter confined between the eyelids so corroding it as to form a thick speck, sufficient to exclude the rays of light. This observation alone, I am confident, if duly attended to, will prevent the blindness of thousands."

He then proceeds to inflammations of the eye produced by blows, wounds, punctures, or acrid substances, and thinks the Egyptian ophthalmia belongs to the last species, and produced by acrid effluvia emanating from persons labouring under the disease.

On the subject of cataract he has bestowed particular attention; he has detailed the causes, symptoms, preventive cure, and operations of depressing and extracting the lens, with a precision and discrimination that, we think, will entitle him to the thanks of junior oculists.

For the other diseases of the organs of vision, we must refer our readers to the work itself; but we think it due to Dr. S. to notice, that he has particularly merited the thanks of the profession for his ingenious suggestions respecting the treatment of specks on the cornea.

